# Socioeconomic inequalities in maternal infections during pregnancy: administrative health data evidence from a large UK urban area with high levels of inequality

**DOI:** 10.1186/s12889-026-27700-x

**Published:** 2026-05-26

**Authors:** Iain Hardie, Kenneth Okelo, Josiah King, Aja Murray, Emily Luedecke, Hildigunnur Anna Hall, Michael V. Lombardo, Louise Marryat, Bonnie Auyeung

**Affiliations:** 1 Department of Psychology, SchoolofPhilosophy,PsychologyandLanguage Sciences, University of Edinburgh, Edinburgh, UK; 2 Centre for Health Security and Communicable Disease Control, Directoratefor Health, Reykjavik, Iceland; 3 Laboratory for Autism and Neurodevelopmental Disorders, Center for Neuroscience and Cognitive Systems, Istituto Itialiano Di Tecnologia, Rovereto, Italy; 4 School of Health Sciences, University of Dundee, Dundee, UK; 5 School of Health and Wellbeing, University of Glasgow, Glasgow, UK

**Keywords:** Prenatal infections, Socioeconomic inequalities, Childhood development

## Abstract

**Background:**

Maternal infections during pregnancy (also known as prenatal infections) have been linked to adverse outcomes such as low birthweight, preterm birth, stillbirth and child developmental concerns, all of which have known socioeconomic inequalities. However, there is currently a lack of research into potential links between socioeconomic factors and prevalence of prenatal infections.

**Methods:**

We utilised linked administrative health data including all children born in a large UK urban area with high levels of inequality, i.e. the National Health Service (NHS) health board of Greater Glasgow & Clyde, Scotland, 2010—2015 (*N* = 92,094). Logistic regression models were used to examine associations between socioeconomic factors (i.e. area-based Scottish Index of Multiple Deprivation (SIMD) quintiles and household-based UK National Statistics Socioeconomic Classification (NS-SEC)), and odds of prenatal infections, measured as hospital-diagnosed prenatal infections and infection-related prescriptions, with and without controlling for covariates.

**Results:**

Area-based deprivation was generally associated with greater odds of prenatal infections, particularly for infections diagnosed in hospital. For example, living in a ‘most deprived’ SIMD quintile area was associated increased odds of both hospital-diagnosed prenatal infections (odds ratio (OR): 1.77; 95% confidence interval (CI): 1.48, 2.11) and infection-related prescriptions during pregnancy (OR: 1.28; 95% CI: 1.21, 1.36), when compared to living in a ‘least deprived’ SIMD quintile area, after covariate adjustment. Meanwhile, lower NS-SEC was also generally associated with increased odds of hospital-diagnosed prenatal infections, but there was not a clear pattern in associations between NS-SEC and infection-related prescriptions.

**Conclusions:**

Area-based deprivation, and to a lesser extent, lower household NS-SEC, appear to be associated with increased odds of maternal infections (particularly hospital-diagnosed maternal infections) during pregnancy in NHS Greater Glasgow & Clyde, Scotland. Socioeconomic inequalities in the prevalence of prenatal infections may therefore be a potential contributing factor to wider inequalities.

**Supplementary Information:**

The online version contains supplementary material available at 10.1186/s12889-026-27700-x.

## Introduction

Maternal infections during pregnancy, also known as ‘prenatal infections’, are commonplace, with around 60% of mothers self-reporting experiencing some form of infection during pregnancy [6]. In addition to contributing to ill-health among mothers, some evidence suggests that prenatal infections are associated with adverse birth outcomes and child developmental outcomes. For example, maternal infections during pregnancy have previously been linked to low birthweight, preterm birth, small for gestational age and stillbirth [1, 7, 22, 23]. Meanwhile, research studies from the United Kingdom (UK), Australia and the United States (US) have also found prenatal infections to be associated with health visitor-reported developmental concerns [12], socioemotional difficulties [11], reduced cognitive skills [17, 19] and a range of other developmental vulnerabilities in the early childhood period [10]. Importantly, research has found that it is particularly severe infections (e.g. infections involving hospitalisation, rather than common/less serious infections), and infections during the second or third trimesters, that are associated with developmental outcomes [12, 17].

In addition to this, there are also known socioeconomic inequalities in adverse birth outcomes and child developmental outcomes. With regards to birth outcomes, research from the UK suggests that pregnancies among those from the most deprived areas have significantly increased odds of low birthweight, preterm birth and stillbirth compared to those from the least deprived areas [38]. Similarly, research from the UK and Ireland also suggests that those from lower levels of socioeconomic status have significantly increased odds of stillbirth, neonatal mortality, perinatal mortality, preterm birth and low birthweight when compared to those from higher levels of socioeconomic status [35].

With regards to child developmental outcomes, analysis of child development data for Scotland suggests that the proportion of children with a developmental concern at their 27—30 month child health review is more than twice as high for those living in the most deprived areas (25.7%) than for those living in the least deprived areas (11.3%) [31]. This is thought to be heavily linked to child poverty, as aspects of living in poverty such as stress, limited money for resources, poorer educational outcomes and poorer employment outcomes can accumulate over time to have increasing impacts on child health and social, emotional and cognitive development [26]. This is supported by further analysis of linked Scottish administrative records, which suggests that children growing up in circumstances of socioeconomic disadvantage, manifested via parental education or occupation, are more likely to experience negative vision-fine-motor, social, and hearing-language developmental outcomes [30]. Similar evidence on this also exists from the rest of the UK and Europe [5, 29].

However, although there are known socioeconomic inequalities in adverse birth outcomes and child developmental outcomes, and potential links between prenatal infections and low birthweight, preterm birth, stillbirth and child developmental outcomes, there is currently a lack of research on whether socioeconomic inequalities exist in the prevalence of prenatal infections. Such research is important as this may be a potential contributing factor to inequalities in birth and child developmental outcomes. Some international literature does exist on the relationship between socioeconomic status and specific types of infections. For example, during the COVID-19 pandemic, a few studies examined socioeconomic inequalities in the prevalence of COVID-19 infections during pregnancy. Some such studies, e.g. Ames et al. [2] which used data on a diverse population of pregnant women in Northern California during the first wave of COVID-19, found the prevalence of infections to be higher among those from high deprivation neighbourhoods, whilst other studies, e.g. Llorca et al. [20] which examined data from Santander, Spain, found housing characteristics but not educational or occupational variables (except where the pregnant woman was a healthcare worker) to be associated with infection prevalence. Other studies have looked at socioeconomic inequalities in the severity of COVID-19 infections, and found lower socioeconomic status and lower educational attainment to be associated with greater odds of developing more severe infections [21].

Outside of COVID-19, there are only a few studies examining socioeconomic inequalities in the prevalence of prenatal infections. For example, Wang et al. [37], who examined economic-related inequalities in hepatitis B virus infection among pregnant women in China, found infections to be more concentrated among those with lower economic status. Similarly, Lantos et al. [18] examined geographic variations in cytomegalovirus infections during pregnancy in Durham, US, and found a high prevalence among those in urban low-income neighborhoods. Finally, one study which examined prenatal infections more generally is Nicholls-Dempsey et al. [27], which studied how socioeconomic status affected pregnancy outcomes (including infections) in the US and found high socioeconomic status to be generally associated with better outcomes.

There are multiple, often overlapping, reasons why those of lower socioeconomic status may be at higher risk of infections, both during pregnancy and more generally. In general, infectious diseases, like many other health issues, can be influenced by both structural determinants (e.g. income, education, employment) and intermediary determinants (e.g. living and working conditions), and these can interact to produce negative outcomes [8]. Data from the UK suggests that socioeconomic status may impact infection risk by affecting individuals’ lifestyle, environmental pollution and chronic comorbidities [39]. Meanwhile, reviews of health inequalities in infectious diseases in the UK and other high-income countries provide evidence that people with lower levels of income, lower educational attainment, unemployment and higher levels of deprivation are at higher risk of infectious diseases and also have lower vaccination uptake [3]. However, few studies to date have examined prevalence of infections specifically among pregnant women. Therefore, in order to provide insight into the role of prenatal infections in health inequalities, more research is required on the socioeconomic patterning of prenatal infections. Our study uses linked administrative health data from the NHS health board of Greater Glasgow & Clyde, Scotland, to examine the extent to which socioeconomic inequalities exist in the prevalence of maternal infections during pregnancy. We specifically address the following research question: are socioeconomic factors, i.e. area-based SIMD quintiles and household-based UK National Statistics Socioeconomic Classification (NS-SEC), associated with odds of maternal infections (measured as hospital-diagnosed infections and receipt of infection-related prescriptions) during pregnancy in NHS Greater Glasgow & Clyde, Scotland?

## Methods

### Setting

The setting for our study, NHS Greater Glasgow & Clyde, Scotland, is a mostly urban area which is the largest health board in Scotland (and one of the largest in the UK). It has a population of around 1.3 million people [25], and it is an area with high levels of child poverty, high levels of deprivation, and large socioeconomic inequalities [36].

### Data and participants

We used linked administrative health data for a cohort of mothers who gave birth in NHS Greater Glasgow & Clyde between 2010 and 2015. This was made up of: (a) mothers’ health records from during their pregnancy, including hospital admissions data from Scottish Morbidity Record (SMR) 01 and 02 (see [13, 14] and prescriptions data from Scotland’s Prescribing Information System (PIS) [15], and (b) birth records from the birth of their child(ren), including additional SMR02 hospital admissions data from the birth as well as formal records from Scottish Birth Records (SBR) [32], and National Records of Scotland (NRS) birth records [24].

In total, there were 92,240 live births in NHS Greater Glasgow & Clyde between 2010 and 2015. Of these, 146 had missing data on SIMD and/or household NS-SEC so were excluded. This meant that 92,094 mothers were included in the final cohort (i.e. 99.8% of all NHS Greater Glasgow & Clyde births recorded during the analysis period).

### Measures

Our analysis included two outcome variables measuring indicators of maternal infections during pregnancy, two explanatory variables measuring SIMD and NS-SEC, and four control variables measuring covariates.

### Outcome variables

The two outcome variables indicating prenatal infection were ‘hospital-diagnosed maternal infection during pregnancy’ and ‘maternal infection-related prescriptions during pregnancy’.

Firstly, hospital-diagnosed maternal infection during pregnancy indicates whether mothers were diagnosed with an infection in hospital during their pregnancy, i.e. in one of the nine months preceding childbirth (coded: 0 = no, 1 = yes). We define infections during pregnancy using International Classification of Diseases 10 (ICD10) codes for infection/inflammation recorded as either the ‘main condition’ or ‘other condition’ (i.e. in cases where more than one diagnosis was made during the same hospital visit) in SMR01 (general hospital inpatient/day case admissions) and SMR02 (maternity hospital inpatient/day case admissions) records. For a full list of the ICD10 codes we included, see Supporting Information Table S1. In addition, for a list of the most common specific four digit ICD10 codes picked up in the measure, see Supporting Information Table S2. Notably, as this measure includes infections that were diagnosed in hospital only, this means that it is likely to have included only more severe infection cases.

Secondly, maternal infection-related prescriptions during pregnancy indicates whether mothers received any prescriptions likely to have been for an infection during their pregnancy. A full list of the drugs we included as infection-related prescriptions is provided in Supporting Information Table S3, and for a list of the most common prescribed drugs picked up in this measure see Supporting Information Table S4. This is a much broader indicator of prenatal infection and is intended to capture a wider range of infections that occurred during pregnancy, including less severe cases (e.g. where individuals visited their general practitioner (GP) but hospital admission was not required).

### Explanatory variables

The two explanatory variables measuring socioeconomic factors were ‘area-based SIMD quintile’ and ‘household-based NS-SEC’.

Firstly, SIMD quintiles measure area-based deprivation levels based on mothers’ home postcodes. Specifically, it indicates the extent to which the area where each mother was resident in was deprived across the following seven domains: income, employment, education, health, access to services, crime and housing. To ensure that SIMD was both: (a) measured temporally before our outcome variables (i.e. so that prenatal infections that were included in our prenatal infection measure could not have influenced the health domain of SIMD measures), and (b) as up—to-date as possible, we used the 2009 version of SIMD for mothers who gave birth in 2010—2011 and the 2012 version of SIMD for mothers who gave birth in 2012—2015 (see [33, 34]. The variable was coded as follows: 1 = ‘most deprived’, 2 = ‘more deprived’, 3 = ‘medium deprived’, 4 = ‘less deprived’, 5 = ‘least deprived’. It is important to note that these quintiles indicate how deprived the area the mother lives in is relative to other areas throughout Scotland (i.e. it is a Scotland-wide measure, not a Greater Glasgow & Clyde specific measure).

Secondly, household NS-SEC is a socioeconomic classification which measures household socioeconomic position based on the occupation and job characteristics of the primary household person [28]. The base data source for this is NRS birth records. NRS defined the primary household person as the father if the birth record was jointly registered by a married couple, and as the mother if the birth record was sole registered or jointly registration by an unmarried couple [24]. We used a four-category version of NS- SEC, which was coded as follows: 1 = not employed (i.e. never worked/long-term unemployed/student/no classifiable occupation), 2 = routine/manual (i.e. routine occupation/semi-routine occupation/lower supervisory or technical occupation), 3 = intermediate (i.e. intermediate occupation/small employer or own account worker), 4 = managerial/professional (i.e. large employer/higher managerial/higher professional/lower managerial or professional).

### Control variables

Our analysis also included four control variables to account for external maternal health and demographic characteristics for which data were available. These were: (a) ‘maternal age’, (b) ‘maternal smoking during pregnancy’, (c) ‘maternal recorded diabetes’, and (d) ‘maternal marital status’. These were selected because they are potentially associated with SIMD, NS-SEC and/or prevalence of prenatal infection, and thus should be controlled for in order to better isolate the associations with prenatal infection. Maternal age is a continuous variable which, using SMR02 maternity hospital data, indicates the mother’s age at the time of childbirth. Maternal smoking during pregnancy and maternal recorded diabetes also both use SMR02 maternity hospital data and indicate whether the mother smoked, or had a record of having diabetes, during the pregnancy (coded: 0 = no, 1 = yes). Finally, maternal marital status is a categorical variable which comes from NRS birth records and indicates marital status at the time of childbirth (coded: 1 = married/cohabiting, 2 = not married/cohabiting but birth jointly registered, 3 = single parent (i.e. birth solo registered).

### Statistical analysis

First, descriptive statistics were gathered to highlight frequencies of prenatal infection variables and control variables across SIMD quintiles and household NS-SEC. Next, logistic regression modelling was used to model unadjusted and covariate-adjusted associations between prenatal infection variables and SIMD quintile/household NS-SEC variables, with covariate-adjusted models additionally adjusting for yearly time-fixed effects. Finally, we then used coefficient plots (see [16] in order to display the results of the modelling graphically.

In addition to the main analysis, we also carried out one piece of sensitivity analysis in which our models relating to NS-SEC were stratified on marital status. This was carried out because (as outlined above in the explanatory variables section) NS-SEC is based on the occupation of the primary household person and this is decided using the (rather arbitrary) definition of being the father if the birth record is jointly registered by a married couple, and as the mother if the birth record is sole registered or jointly registration by an unmarried couple. Therefore, stratifying the analysis by marital status is a way of testing if this may impact our results.

All analysis was conducted using Stata/IC 16.1 software within Scotland’s National Safe Haven, where access to the data was coordinated by Public Health Scotland’s Electronic Data Research and Innovation Service (eDRIS).

## Results

### Descriptive statistics

The descriptive statistics showing frequencies of our analysis variables by SIMD quintile and household NS-SEC are provided in Table 1.

**Table 1:** Descriptive statistics for prenatal infections and control variables by SIMD quintile and NS-SEC

	**N (% of Total)**									
		**SIMD Quintile**					**NS-SEC**			
		**1 (Most deprived)**	**2 (More deprived)**	**3 (Medium deprived)**	**4 (Less deprived)**	**5 (Least deprived)**	**Not Employed**	**Routine/Manual**	**Intermediate**	**Managerial/Professional**
	92,094 (100%)	36,732 (39.9%)	17,311 (18.8%)	14,113 (15.3%)	11,562 (12.6%)	12,376 (13.4%)	17,121 (18.6%)	28,516 (30.9%)	17,744 (19.3%)	28,713 (31.2%)
**Prenatal Infections**										
Hospital-diagnosed prenatal infection(s)										
*No*	89,565 (97.3%)	35,434 (96.5%)	16,818 (97.1%)	13,771 (97.6%)	11,322 (97.9%)	12,220 (98.7%)	16,425 (95.9%)	27,621 (96.9%)	17,283 (97.4%)	28,236 (98.3%)
*Yes*	2,529 (2.7%)	1,298 (3.5%)	493 (2.9%)	342 (2.4%)	240 (2.1%)	156 (1.3%)	696 (4.1%)	895 (3.1%)	461 (2.6%)	477 (1.7%)
Infection-related prescription(s) during pregnancy										
*No*	70,816 (76.9%)	27,109 (73.8%)	13,143 (75.9%)	11,022 (78.1%)	9,258 (80.1%)	10,284 (83.1%)	12,749 (74.5%)	21,402 (75.1%)	13,523 (76.2%)	23,142 (80.6%)
*Yes*	21,278 (23.1%)	9,623 (26.2%)	4,168 (24.1%)	3,091 (21.9%)	2,304 (19.9%)	2,092 (16.9%)	4,372 (25.5%)	7,114 (24.9%)	4,221 (23.8%)	5,571 (19.4%)
**Control Variables**										
Maternal age (mean)	29.5 years	27.7 years	29.1 years	30.2 years	31.4 years	32.7 years	25.4 years	28.9 years	29.9 years	32.3 years
Maternal smoking during pregnancy										
*No*	78,671 (85.4%)	28,247 (76.9%)	14,820 (85.6%)	12,672 (89.8%)	10,887 (94.2%)	12,045 (97.3%)	11,736 (68.5%)	23,341 (81.9%)	16,045 (90.4%)	27,549 (95.9%)
*Yes*	13,423 (14.6%)	8,485 (23.1%)	2,491 (14.4%)	1,441 (10.2%)	675 (5.8%)	331 (2.7%)	5,385 (31.5%)	5,175 (18.1%)	1,699 (9.6%)	1,164 (4.1%)
Maternal recorded diabetes										
*No*	89,854 (97.6%)	35,786 (97.4%)	16,889 (97.6%)	13,769 (97.6%)	11,241 (97.2%)	12,169 (98.3%)	16,731 (97.7%)	27,768 (97.4%)	17,273 (97.4%)	28,082 (97.8%)
*Yes*	2,240 (2.4%)	946 (2.6%)	422 (2.4%)	344 (2.4%)	321 (2.8%)	207 (1.7%)	390 (2.3%)	748 (2.7%)	471 (2.7%)	631 (2.2%)
Maternal marital status										
*Married/cohabiting*	72,675 (78.9%)	24,609 (67.0%)	13,721 (79.3%)	12,139 (86.0%)	10,454 (90.4%)	11,752 (95.0%)	8,936 (52.2%)	21,829 (76,6%)	14,834 (83.6%)	27,076 (94.3%)
* Not married/cohabiting but birth jointly registered*	14,391 (15.6%)	9,009 (24.5%)	2,664 (15.4%)	1,461 (10.4%)	810 (7.0%)	447 (3.6%)	5,659 (33.1%)	5,206 (18.3%)	2,285 (12.9%)	1,241 (4.3%)
*Single parent (birth solo registered)*	5,028 (5.5%)	3,114 (8.5%)	926 (5.3%)	513 (3.6%)	298 (2.6%)	177 (1.43%)	2,526 (14.7%)	1,481 (5.2%)	625 (3.5%)	396 (1.4%)

Many of the mothers in the study were from more deprived areas, with 39.9% being from a ‘most deprived’ SIMD quintile area. This reflects the fact that many of Scotland’s most deprived areas are in Glasgow [36]. Overall, 2.7% of the mothers were diagnosed with an infection in hospital during pregnancy, whilst 23.1% received an infection-related prescription during pregnancy. Notably, the prevalence of these prenatal infection variables appeared to be higher for those from more deprived areas and with lower NS-SEC. For example, 3.5% of those from the ‘most deprived’ SIMD quintile areas had hospital-diagnosed prenatal infections compared to 1.3% of those in the ‘least deprived’ ones. Similarly, 4.1% of those who were not employed had hospital-diagnosed prenatal infections compared to 1.7% of those who were employed in managerial/professional occupations.

With regards to the other variables, the mean age of mothers at the time of childbirth was 29.5 years, and maternal age generally tended to be higher for those who were living in less deprived areas or had higher household NS-SEC. Meanwhile, 14.6% of mothers smoked during their pregnancy and this also had a strong social gradient. E.g., 23.1% of those in the most deprived areas smoked compared to just 2.7% of those in the least deprived areas. 2.4% of mothers in the sample had diabetes, and this was slightly lower for those in less deprived areas. Finally, 78.9% of mothers in the sample were married or cohabiting at the time of childbirth, whilst 15.6% were not married/cohabiting but jointly registered the birth and 5.5% registered the birth as a single parent. In general, those living in less deprived areas and of higher household NS-SEC were more likely to be married/cohabiting.

### Modelling results on relationship between socioeconomic factors and odds of prenatal infection

**Fig. 1:**
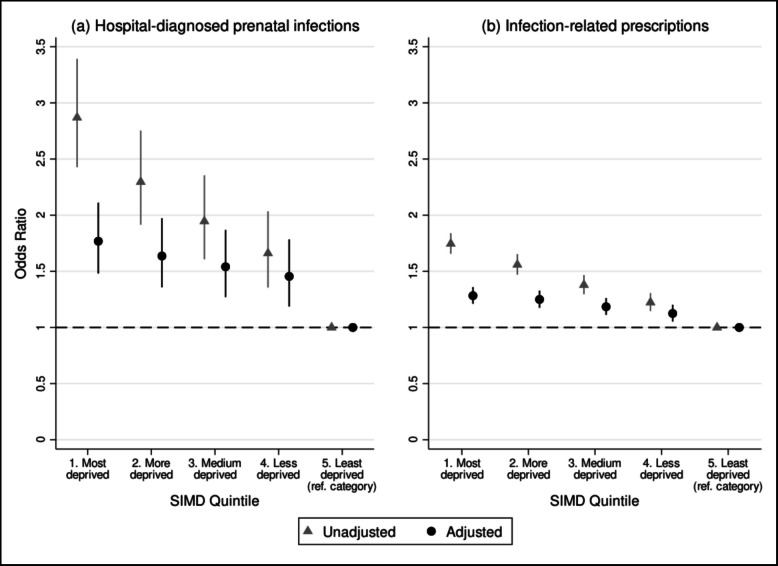
Odds Ratios (and 95% CIs) showing unadjusted and covariate-adjusted associations between area-based SIMD quintiles, hospital-diagnosed prenatal infections and infection-related prescriptions during pregnancy. *Notes*: adjusted models adjust for maternal age, smoking status, recorded diabetes and marital status. Odds ratios are represented by the circular/triangular points and 95% confidence intervals are represented by the vertical bars

**Fig. 2:**
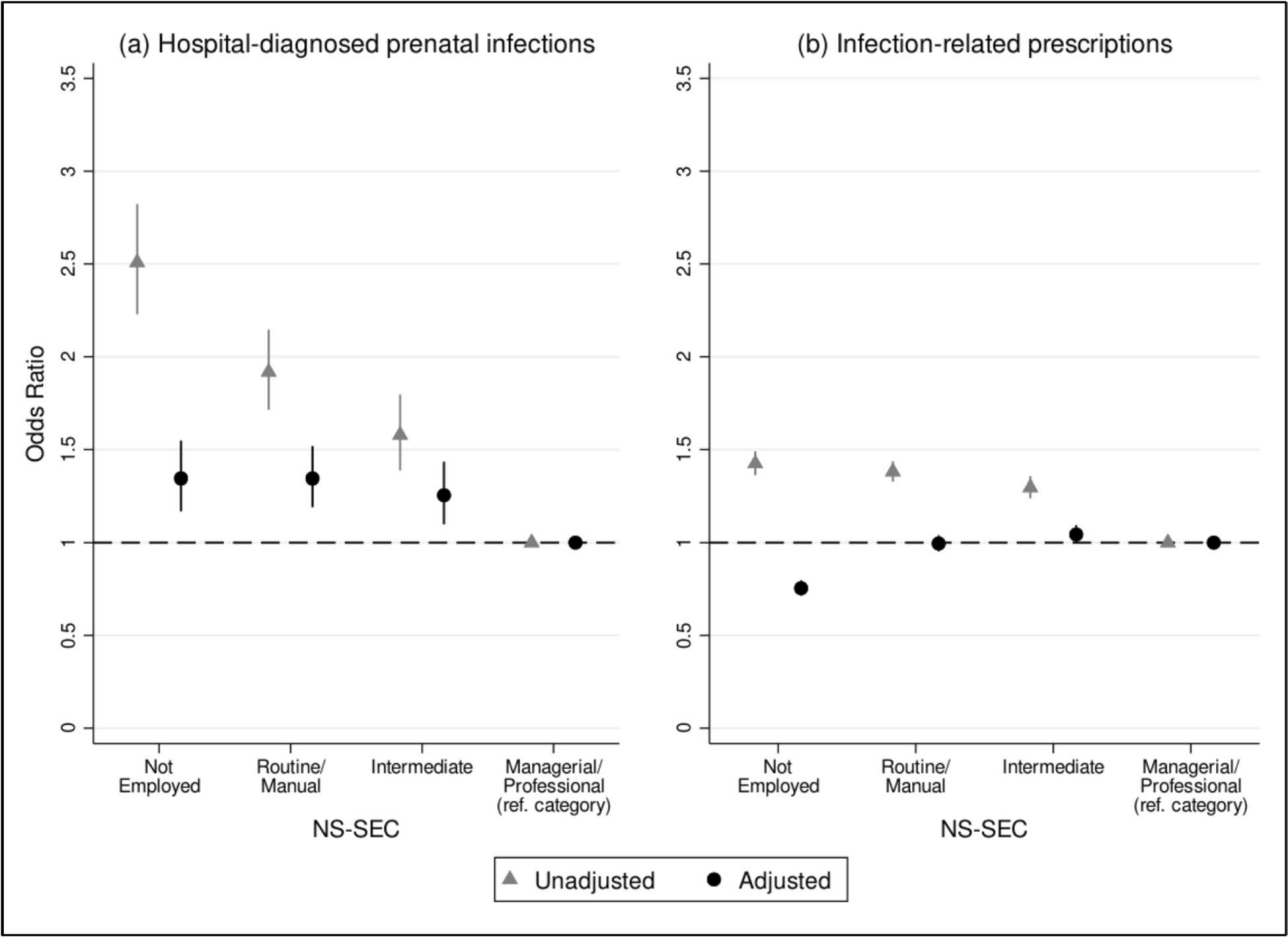
Odds Ratios (and 95% CIs) showing unadjusted and covariate-adjusted associations between household-based NS-SEC, hospital-diagnosed prenatal infections and infection-related prescriptions during pregnancy. *Notes*: adjusted models adjust for maternal age, smoking status, recorded diabetes and marital status. Odds ratios are represented by the circular/triangular points and 95% confidence intervals are represented by the vertical bar

The results of the regression models examining unadjusted and adjusted relationships between socioeconomic factors and odds of prenatal infections are provided visually in Figure 1 (for area-based SIMD quintiles) and Figure 2 (for household NS-SEC), with full detailed results provided in table form in Supporting Information Table S5.

Firstly, the results suggest that area-based deprivation was generally associated with greater odds of hospital-diagnosed prenatal infections. For example, compared to living in a ‘least deprived’ SIMD quintile area, living in a ‘most deprived’ quintile area was associated with increased odds of hospital-diagnosed prenatal infections, even after covariate adjustment (odds ratio (OR): 1.77; 95% confidence interval (CI): 1.48, 2.11). There were similar results for infection related prescriptions, although effect sizes were smaller (OR: 1.28; 95% CI: 1.21, 1.36).

Household NS-SEC was also associated with significant differences in odds of hospital-diagnosed prenatal infections. Those who were not employed (OR: 1.35; 95% CI: 1.17, 1.55), had routine/manual occupations (OR: 1.34; 95% CI: 1.19, 1.52) and had intermediate occupations (OR: 1.26; 95% CI; 1.19; 1.43) all had increased odds of hospital-diagnosed prenatal infections compared to those in managerial/professional occupations, after covariate adjustment. However, results for infection-related prescriptions were less clear. There were no significant associations except for that those who were not employed had reduced odds of infection-related prescriptions (OR: 0.75; 0.71, 0.80) compared to those in managerial/professional occupations after covariate adjustment.

### Sensitivity analysis

The results of the sensitivity analysis in which models were stratified by marital status are provided in Supporting Information Table S6. Results were broadly similar to the main analysis for those who were both unmarried and married.

## Discussion

This study examined socioeconomic inequalities in the odds of maternal infections during pregnancy by making use of linked administrative health data on the mothers of children born in NHS Greater Glasgow & Clyde, Scotland, between 2010 and 2015. Overall, our findings suggest that socioeconomic inequalities in maternal infections during pregnancy do appear to exist.

Socioeconomic inequalities were particularly apparent for hospital-diagnosed prenatal infections, i.e. infections that were likely more severe, whereas effect sizes were smaller when using the broader infection-related prescription measure. This may reflect those in deprived areas and of lower household NS-SEC being more susceptible to severe infections due to being more likely to have pre-existing health problems or poorer health outcomes related to having lower incomes or living in poverty [4]. Indeed, previous research has found links between socioeconomic deprivation, low socioeconomic status and the severity of certain types of infection or infection-related complications [9, 40].

Our findings also highlight that socioeconomic inequalities were clearer for our area-based deprivation measure (SIMD) than for our household NS-SEC measure. For example, effect sizes of associations between SIMD quintile and hospital-diagnosed prenatal infections were larger than effect sizes for associations between household NS-SEC and hospital-diagnosed prenatal infections. Moreover, the pattern of the relationship between household NS-SEC and infection-related prescriptions during pregnancy was less clear than for SIMD quintiles. E.g., one perhaps surprising finding was those who were ‘not employed’ appeared to have decreased odds of infection-related prescriptions compared to those in ‘managerial/professional’ occupations, after controlling for covariates. This may reflect the fact that SIMD takes into account a wider range of factors (i.e. income, employment, education, health, access to services, crime and housing). NS-SEC, on the other hand, takes into account occupation only and the ‘not employed’ category for example will include people in a wide range of different situations (from long-term unemployed people who are in very disadvantaged position to full-time students who may be less disadvantaged e.g. if they come from a wealthier background). It may also be the case that people who are not employed are simply coming into contact with fewer people (due to not being in the workplace or on public transport during rush hour, for example), which makes them less likely to pick up certain types of infections.

These findings highlight that inequalities in the prevalence of infections during pregnancy may contribute to wider health inequalities. As such, interventions targeted at prevention of key infections, particularly those highlighted in Supporting Information Table S2 as the most common hospital-diagnosed infections during pregnancy, e.g. urinary/genitourinary tract infections, respiratory/lower respiratory infections and kidney infections, may generally help reduce inequalities.

Moreover, in addition to contributing to ill-health and health inequalities generally, possible additional implications of our findings relate to adverse birth outcomes and childhood developmental outcomes. Whilst our study did not include any analysis of impacts on birth outcomes or childhood developmental outcomes (and so cannot make any conclusions around this based on our analysis), it is possible that the socioeconomic inequalities that we observe in prenatal infections may contribute to the socioeconomic inequalities that are known to exist in adverse birth outcomes and child developmental outcomes in Scotland [30, 31] and the rest of the UK/elsewhere [5, 29, 35, 38]. This is because prenatal infections are associated with adverse birth outcomes [1, 7, 22, 23] and child developmental outcomes [10, 11, 12, 17, 19], so it may potentially be the case that additional prenatal infections that are occurring for those from deprived areas is one of the reasons that they experience these other poorer outcomes. More research (specifically research testing whether prenatal infections may mediate the relationship between socioeconomic status and these outcomes) is required to confirm whether this is the case or not.

The key strength of the present study is that it was able to make use of a very large linked administrative health dataset which covered the mothers of almost all children born in NHS Greater Glasgow & Clyde, Scotland between 2010 and 2015 (92,094, or 99.8%, of the 92,240 live births recorded in the area during this period were included in our analysis).

However, there are also some notable limitations. Firstly, we were unable to control for factors like air pollution, maternal nutrition and maternal BMI in our analysis, which could be associated with both socioeconomic factors and prenatal infections. Secondly, we do not have data on which postcode or datazone individuals are from, meaning it was not possible to control for any potential spatial dependency. Thirdly, our explanatory variables do not perfectly capture deprivation or socioeconomic status. For example, although SIMD quintiles take into account deprivation across a range of domains, it is limited by being area-based rather than individual level, while household NS-SEC is limited in that it covers occupation only and not other aspects of socioeconomic status like education or income. Fourthly, our outcome variables do not perfectly capture maternal infections during pregnancy. The hospital-diagnosed prenatal infections measure likely includes only a small proportion of all infections during pregnancy (i.e. the cases severe enough to involve hospitalisation). Whilst the broader infection-related prescriptions measure will capture a much wider range of infections it is only a proxy measure for infection. Ideally, other sources of infection information, such as GP records, would have been used to examine less severe cases. However, GP data was not available for our study.

There are also some broader limitations of using the type of administrative health data used in our analysis. Importantly, our hospital-diagnosed infections and infection-related prescription variables are essentially measuring contact with healthcare systems due to infection (rather than capturing all infections) and it is possible that health-seeking behaviour may be socially patterned, which could affect our results. It is also possible that our data may be influenced by variations in prescribing practices between different GPs or GP practices, or variations in prescribing practices over time.

## Conclusions

To conclude, our study, despite its limitations, provides important evidence on socioeconomic inequalities in maternal infections during pregnancy. In particular, we highlight that deprivation and, to a lesser extent, low household NS-SEC, are associated with increased odds of maternal infections during pregnancy in NHS Greater Glasgow & Clyde, Scotland. Future research could test whether this may be a potential contributing factor to wider inequalities.

## Supplementary Information

Supplementary Material 1.

## Data Availability

The administrative health datasets used for this study are not publicly available, but can be accessed via successfully applying to the NHS HSC-PBPP. The authors of the present study were supported in applying for approval from HSC-PBPP by the eDRIS team at Public Health Scotland. eDRIS also facilitated access to the data via Scotland’s National Safe Haven.

## Abbreviations

SIMD: Scottish Index of Multiple Deprivation
NS-SEC: National Statistics Socioeconomic Classification
UK: United Kingdom
US: United States
ADHD: Attention Deficit Hyperactivity Disorder
MIA: Maternal Immune Activation
NHS: National Health Service
PIS: Prescribing Information System
SMR: Scottish Morbidity Record
NRS: National Records for Scotland
ICD: International Classification of Diseases
GP: General Practitioner
eDRIS: Electronic Data Research and Innovation Service
HSC-PBPP: Public Benefit and Privacy Panel for Health and Social Care
