# Supplementary material for: Socioeconomic inequalities in maternal infections during pregnancy: administrative health data evidence from a large UK urban area with high levels of inequality
Source: BMC Public Health. 2026 May 26;26:2193. doi: 10.1186/s12889-026-27700-x (PMC13390274; doi:10.1186/s12889-026-27700-x)
Supplement: Supplementary file 1 — Supplementary Material 1. [file 12889_2026_27700_MOESM1_ESM.docx]

**Table S1. ICD10 infection codes included in hospital-diagnosed maternal infections during pregnancy measure**

| ICD10 category | **List of infections included and their specific ICD10 codes** |
| --- | --- |
| Certain infectious & parasitic diseases | Intestinal infectious diseases (A00-A09); Tuberculosis (A15-A19); Certain zoonotic bacterial diseases (A20-A28); Other bacterial diseases (A30-A49); Infections with a predominantly sexual mode of transmission (A50-A64); Other spirochaetal diseases (A65-A69); Other diseases caused by chlamydiae (A70-A74); Rickettsioses (A75-A79); Viral infections of the central nervous system (A80-A89); Arthropod-borne viral fevers and viral haemorrhagic fevers (A92-A99); Viral infections characterized by skin and mucous membrane lesions (B00-B09); Viral hepatitis (B15-B19); Human immunodeficiency virus [HIV] disease (B20-B24); Other viral diseases (B25-B34); Mycoses (B35-B49); Protozoal diseases (B50-B64); Helminthiases (B65- B83); Pediculosis (B85-B89); acariasis and other infestations (B85-B89); Sequelae of  infectious/parasitic diseases (B90-B94); Bacterial, viral and other infectious agents (B95-B98); Other infectious diseases (B99) |
| Diseases of the nervous system | Inflammatory diseases of the central nervous system (G00-G09); Multiple cranial nerve palsies in infectious/parasitic diseases (G531); Polyneuropathy in infectious/parasitic diseases (G630);  Hydrocephalus in infectious/parasitic diseases (G940) |
| Diseases of the eye and adnexa | Parasitic infestation of eyelid (H030); Involvement of eyelid in other infectious diseases (H031); Conjunctivitis (H1); Iridocyclitis in infectious/parasitic diseases (H220); Chorioretinal inflammation in infectious/parasitic diseases (H320); Purulent endophthalmitis (H440); Other endophthalmitis (H441); Retrobulbar neuritis in diseases (H481) |
| Diseases of the ear and mastoid process | Otitis externa (H60 & H62); Otitis media (H66-H67); Eustachian salpingitis (H68); Mastoiditis and related conditions (H70); Myringitis (H730-H731); Mastoiditis in infectious/parasitic diseases (H750); Acoustic neuritis in infectious/parasitic diseases (H940) |
| Diseases of the circulatory system | Pericarditis (I30, I310, I311 and I32); Acute and subacute infective endocarditis (I33, I38 and I39); Myocarditis (I40 and I41); Cardiomyopathy in infectious/parasitic diseases (I430); heart disorders in bacterial diseases (I520); Other heart disorders in other infectious/parasitic diseases (I521); Cerebral arteritis in infectious/parasitic diseases (I681); Cardiovascular  syphilis (I980); Cardiovascular disorders in other infectious/parasitic diseases (I981) |
| Diseases of the respiratory system | Acute upper respiratory infections (J00-J06); Influenza and pneumonia (J09-J18); Other acute lower respiratory infections (J20-J22); Chronic rhinitis, nasopharyngitis and pharyngitis (J31) Chronic sinusitis (J32); Chronic tonsilitis (J350); Peritonsillar abscess (J36); Chronic laryngitis and laryngotracheitis (J37); Other infection of upper respiratory tract (J39); Bronchitis (J40- J42); Chronic obstructive pulmonary disease with acute lower respiratory infection (J440);  Abscess of lung and mediastinum (J85); Pyothorax (J86) |
| Diseases of the digestive system | Pulpitis (K040); Periodontitis (K044-K045); Gingivitis and Periodontitis (K05); Sialoadenitis (K112); Abscess of salivary gland (K113); Cellulitis and abscess of mouth (K122); Glossitis (K140); Appendicitis (K35); Abscess of anal and rectal regions (K61); Abscess of intestine (K630); Peritonitis (K65); Disorders of peritoneum in infectious diseases (K67); Liver disorders in infectious/parasitic diseases (K770); Tuberculous disorders of intestines, peritoneum and  mesenteric glands (K930) |
| Diseases of the skin | Infections of the skin (and subcutaneous tissue) (L00-L08) |
| Diseases of the musculoskeletal system | Infectious Arthropathies (M00-M03) |
| genitourinary system | Glomerular disorders in infectious/parasitic diseases (N08); tubulo-interstitial nephritis (N10- N12); Late syphilis of kidney (N290); Other disorders of kidney and ureter in infectious/parasitic diseases (N291); Cystitis (N30 and N330); Urethritis (N34); Urinary tract infection (N390);  Inflammatory disorders of breast (N61); Inflammatory diseases of female pelvic organs (N7); |
| Pregnancy and childbirth | Infections of genitourinary tract in pregnancy (O23); Maternal infectious/parasitic diseases complicating pregnancy (O98) |
| Consequences of external causes | Infections following infusion, transfusion and therapeutic injection (T802); Infection following a procedure (T814); Infection and inflammatory reaction due to cardiac valve prosthesis or other cardiac and vascular devices, implants and grafts (T826-T827); Infection and inflammatory reaction due to prosthetic device, implant and graft (T835-T836); Infection and inflammatory reaction due to internal joint prosthesis or other device (T845-T847); Infection and inflammatory reaction due to other internal prosthetic devices, implants and grafts (T857); Infection of  amputation stump (T6874); Infection following immunisation (T880) |

**Table S2. List of most common ICD10 infection codes picked up in hospital-diagnosed maternal infections during pregnancy measure**

| **ICD10 Code** | **Description** | **Number of recorded infections** |
| --- | --- | --- |
| 0234 | Unspecified infection of urinary tract in pregnancy | 477 |
| 0239 | Other and unspecified genitourinary tract infection in pregnancy | 400 |
| N390 | Urinary tract infection, site not specified | 257 |
| J22X | Unspecified acute lower respiratory infection | 236 |
| 0235 | Infections of the genital tract in pregnancy | 164 |
| 0230 | Infections of kidney in pregnancy | 111 |
| N771 | Vaginitis, vulvitis and vulvovaginitis in infectious and parasitic diseases | 83 |
| B373 | Candidiasis of vulva and vagina | 78 |
| B349 | Viral infection, unspecified | 70 |
| B951 | Streptococcus group B | 62 |
| B962 | Escherichia coli [E.coli] | 62 |
| A099 | Gastroenteritis and colitis of unspecified origin | 55 |
| L022 | Cutaneous abscess, furuncle and carbuncle of trunk | 40 |
| A09X | Other gastroenteritis and colitis of infectious and unspecified origin | 35 |
| A491 | Streptoccoccal and enterococcal infection, unspecified site | 30 |
| J039 | Acute tonsillitis | 26 |
| J069 | Acute upper respiratory infection, site unspecified | 25 |
| L050 | Pilonidal cyst with abscess | 24 |
| K610 | Anal abscess | 22 |
| A084 | Viral intestinal infection, unspecified | 20 |
| B379 | Candiasis, unspecified | 18 |
| A153 | Tuberculosis of lung, confirmed by unspecified means | 17 |

**Table S3. Prescribed drugs included in infection-related prescriptions during pregnancy measure**

| **Drug category** | **List of prescribed drugs included** |
| --- | --- |
| Antibacterial Drugs | - Penicillin (Amoxicillin, Ampicillin, Co-amoxiclav, Co-fluampicil, Flucloxacillin, Phenoxymethylpenicillin) - Cephalosporins and other beta-lactams (Cefaclor, Cephalexin, Cefixime, Cefradine, Cefuroxime) - Tetracyclines (Doxycycline, Lymecycline, Minocycline, Oxytetracycline, Clobetasone with Oxytetracycline and Nystatin, Tretracycline) - Aminoglycosides (Gentamicin, Neomycin Sulfate, Betamethasone with Neomycin, Hydrocortisone with Neomycin, Triamcinolone Gramicidin Neoymycin and Nystatin, Dexamethasone with Neomycin and Polymyxin B) - Macrolides (Azithromycin, Clarithromycin, Erythromycin, Erythromycin with Zinc Acetate, Isotretinoin with Erythromycin) - Clindamycin and Lincomycin (clindamycin) - Other Antibacterials (Chloramphenicol, Colistin, Fusidic Acid, Betamethason with Fusidic Acid, Hydrocortisone with Fusidic Acid, Vancomycin) - Antituberculosis Drugs (Cycloserine, Ethambutol Hydrochloride, Rifampicin with Isoniazid, Rifampicin) - Antileprotic Drugs (Dapsone) - Metronidazole, Tinidazole and Ornidazole (Metronidazole, Tinidazole) - Quinolones (Cifrofloxacin, Levofloxacin, Moxifloxacin, Norfloxacin, Ofloxacin) - Urinary Tract Infection Drugs (Methenamine, Nitrofurantoin) |
| Antifungal Drugs | - Triazole Antifungals (Fluconazole, Fluconozole and Clotrimazole, Itraconazole) - Imidazole Antifungals (Ketoconazole) - Polyene Antifungals (Amphotericin, Nystatin) - Other Antifungals (Griseofulvin) |
| Antiviral Drugs | - Herpesvirus Infections (Aciclovir, Famciclovir, Penciclovir, Valaciclovir) - Viral Hepatitis (Enteclavir) - Influenza (Amantadine Hydrochloride, Oseltamivir, Zanamivir) |
| Antiprotozoal Drugs | - Antimalarial drugs (Mefloquine, Quinine, Proguanil Hydrochloride with - Chloroquine Phosphate, Atovaquone with Proguanil Hydrochloride) |
| Anthelmintic Drugs | - Drugs for threadworms (Mebendazole, Piperazine with Senna) |

**Table S4. Most common prescribed drugs picked up in infection-related prescriptions during pregnancy measure**

| **Drug category** | **Prescribed drug** | **Number of prescription receipts recorded** |
| --- | --- | --- |
| Antibacterial drugs | Amoxicillin | 11,276 |
| Antibacterial drugs | Cefalexin | 5,356 |
| Antibacterial drugs | Nitrofurantoin | 3,777 |
| Antibacterial drugs | Flucloxacillin | 2,072 |
| Antibacterial drugs | Metronidazole | 1,853 |
| Antibacterial drugs | Erythromycin | 1,513 |
| Antiviral drugs | Aciclovir | 1,503 |
| Antibacterial drugs | Phenoxymethylpenicillin | 1,397 |
| Antibacterial drugs | Co-amoxiclav | 1,294 |
| Antibacterial drugs | Fusidic acid | 1,180 |
| Antibacterial drugs | Erythromycin with zinc acetate | 1,072 |
| Antibacterial drugs | Betamethasone with fusidic acid | 911 |
| Antibacterial drugs | Neomycin sulfate | 844 |
| Antibacterial drugs | Chloramphenicol | 631 |
| Antibacterial drugs | Hydrocortisone with fusidic acid | 505 |
| Antifungal drugs | Ketocanazole | 491 |
| Antifungal drugs | Flucanazole | 488 |
| Antibacterial drugs | Clindamycin | 432 |

Notes: figures are the total number of prescriptions recorded during pregnancies relating to children included in our study. Cases where a mother was prescribed the same drug more than once during the same pregnancy are counted as separate prescriptions.

**Table S5. Odds ratios (95% CIs) showing associations between area-based SIMD quintiles, household NS-SEC and prenatal infections**

|  | **OR (95% CI)** | | | |
| --- | --- | --- | --- | --- |
|  | **Prenatal Infection(s)** | | | |
|  | **Hospital-diagnosed prenatal infection(s)** | | **Infection-related prescription(s)** | |
|  | **Unadjusted** | **Adjusted** | **Unadjusted** | **Adjusted** |
| **SIMD quintile** |  |  |  |  |
| *1 (Most deprived)* | 2.87***  (2.43, 3.39) | 1.77***  (1.48, 2.11) | 1.75***  (1.66, 1.84) | 1.28***  (1.21, 1.36) |
| *2 (More deprived)* | 2.30***  (1.92, 2.75) | 1.64***  (1.36, 1.97) | 1.56***  (1.47, 1.65) | 1.25***  (1.18, 1.33) |
| *3 (Medium deprived)* | 1.95***  (1.61, 2.35) | 1.54***  (1.19, 1.78) | 1.38***  (1.30, 1.47) | 1.18***  (1.11, 1.26) |
| *4 (Less deprived)* | 1.66***  (1.36, 2.03) | 1.45***  (1.19, 1.78) | 1.22***  (1.15, 1.31) | 1.12***  (1.05, 1.20) |
| *[5 (Least deprived)]* |  |  |  |  |
| **NS-SEC** |  |  |  |  |
| *Not Employed* | 2.51***  (2.23, 2.82) | 1.35***  (1.17, 1.55) | 1.42***  (1.36, 1.49) | 0.75***  (0.71, 0.80) |
| *Routine/Manual* | 1.92***  (1.71, 2.15) | 1.34***  (1.19, 1.52) | 1.38***  (1.33, 1.44) | 1.00  (0.95, 1.04) |
| *Intermediate* | 1.58***  (1.39, 1.80) | 1.26**  (1.10, 1.43) | 1.30*** (1.24, 1.36) | 1.04  (1.00, 1.09) |
| *[Managerial/Professional]* |  |  |  |  |
| **Maternal age** |  | 0.96***  (0.95, 0.97) |  | 0.95***  (0.95, 0.95) |
| **Maternal smoking during pregnancy** |  |  |  |  |
| *[No]* |  |  |  |  |
| *Yes* |  | 1.28***  (1.15, 1.42) |  | 1.05*  (1.00, 1.09) |
| **Maternal recorded diabetes** |  |  |  |  |
| *[No]* |  |  |  |  |
| *Yes* |  | 2.00***  (1.67, 2.48) |  | 1.24***  (1.12, 1.37) |
| **Maternal marital status** |  |  |  |  |
| *[Married/cohabiting]* |  |  |  |  |
| *Not married/cohabiting but birth jointly registered* |  | 1.35***  (1.21, 1.50) |  | 1.44***  (1.38, 1.51) |
| *Single parent (birth solo registered)* |  | 1.30**  (1.11, 1.51) |  | 1.59***  (1.49, 1.70) |

*Notes:* In unadjusted models, SIMD quintile and NS-SEC are modelled separately. Adjusted models include (yearly) time fixed effects based on the child’s year of birth. Reference categories shown in square brackets. *p<0.05, **p<0.01, ***p<0.001.

**Table S6. Sensitivity analysis: odds ratios (95% CIs) showing associations between NS-SEC and prenatal infections, stratified by marital status**

|  | **OR (95% CI)** | | | | | | | |
| --- | --- | --- | --- | --- | --- | --- | --- | --- |
|  | **Prenatal Infection(s)** | | | | | | | |
|  | **Hospital-diagnosed prenatal infection(s)** | | | | **Infection-related prescription(s)** | | | |
|  | **Married/cohabiting** | | **Not married/cohabiting** | | **Married/cohabiting** | | **Not married/cohabiting** | |
|  | Unadjusted | Adjusted | Unadjusted | Adjusted | Unadjusted | Adjusted | Unadjusted | Adjusted |
| **NS-SEC** |  |  |  |  |  |  |  |  |
| *Not Employed* | 2.21***  (1.91, 2.57) | 1.31**  (1.10, 1.56) | 1.68**  (1.24, 2.29) | 1.41*  (1.02, 1.95) | 1.21***  (1.14, 1.28) | 0.79***  (0.74, 0.85) | 0.82**  (0.73, 0.92) | 0.62***  (0.55, 0.70) |
| *Routine/Manual* | 1.75***  (1.54, 1.98) | 1.29***  (1.13, 1.48) | 1.57**  (1.15, 2.14) | 1.42*  (1.03, 1.95) | 1.27***  (1.22, 1.32) | 1.02  (0.97, 1.06) | 0.96  (0.85, 1.07) | 0.81***  (0.72, 0.91) |
| *Intermediate* | 1.44***  (1.24, 1.65) | 1.20*  (1.03, 1.39) | 1.52*  (1.08, 2.13) | 1.39^+^  (0.98, 1.96) | 1.21***  (1.14, 1.26) | 1.04  (0.99, 1.09) | 1.07  (0.95, 1.22) | 0.93  (0.81, 1.06) |
| *[Managerial/Professional]* |  |  |  |  |  |  |  |  |
| **SIMD quintile** |  |  |  |  |  |  |  |  |
| *1 (Most deprived)* |  | 1.83***  (1.51, 2.22) |  | 1.40  (0.88, 2.23) |  | 1.33***  (1.25, 1.41) |  | 0.83*  (0.70, 0.99) |
| *2 (More deprived)* |  | 1.69***  (1.38, 2.08) |  | 1.28  (0.79, 2.09) |  | 1.28***  (1.19, 1.36) |  | 0.84^+^  (0.70, 1.01) |
| *3 (Medium deprived)* |  | 1.54*** (1.25, 1.90) |  | 1.33  (0.80, 2.22) |  | 1.23***  (1.15, 1.31) |  | 0.75**  (0.62, 0.91) |
| *4 (Less deprived)* |  | 1.36**  (1.08, 1.70) |  | 1.73*  (1.02, 2.93) |  | 1.14***  (1.06, 1.23) |  | 0.82  (0.66, 1.01) |
| *[5 (Least deprived)]* |  |  |  |  |  |  |  |  |
| **Maternal age** |  | 0.96***  (0.95, 0.97) |  | 0.97***  (0.96, 0.98) |  | 0.95***  (0.95, 0.95) |  | 0.95***  (0.94, 0.96) |
| **Maternal smoking during pregnancy** |  |  |  |  |  |  |  |  |
| *[No]* |  |  |  |  |  |  |  |  |
| *Yes* |  | 1.50***  (1.31, 1.72) |  | 1.05  (0.91, 1.22) |  | 1.10**  (1.04, 1.17) |  | 0.99  (0.92, 1.06) |
| **Maternal recorded diabetes** |  |  |  |  |  |  |  |  |
| *[No]* |  |  |  |  |  |  |  |  |
| *Yes* |  | 1.96***  (1.55, 2.49) |  | 2.23***  (1.55, 3.21) |  | 1.25***  (1.12, 1.40) |  | 1.17  (0.94, 1.47) |

*Notes:* In unadjusted models, SIMD quintile and NS-SEC and modelled separately. Adjusted models include (yearly) time fixed effects based on the child’s year of birth. Reference categories shown in square brackets. *p<0.05, **p<0.01, ***p<0.001.
